# Corrigendum: Composition and Diversity of CRISPR-Cas13a Systems in the Genus *Leptotrichia*

**DOI:** 10.3389/fmicb.2020.00179

**Published:** 2020-02-12

**Authors:** Shinya Watanabe, Bintao Cui, Kotaro Kiga, Yoshifumi Aiba, Xin-Ee Tan, Yusuke Sato'o, Moriyuki Kawauchi, Tanit Boonsiri, Kanate Thitiananpakorn, Yusuke Taki, Fen-Yu Li, Aa Haeruman Azam, Yumi Nakada, Teppei Sasahara, Longzhu Cui

**Affiliations:** ^1^Division of Bacteriology, Department of Infection and Immunity, Faculty of Medicine, Jichi Medical University, Tochigi, Japan; ^2^Division of Clinical Laboratory, Tottori University Hospital, Tottori, Japan

**Keywords:** *Leptotrichia*, CRISPR-Cas13a, clustered regularly interspaced short palindromic repeats, CRISPR-Cas, C2c2, crRNA, protospacer, self-targeting spacer

In the original article, there was a mistake in Table 1 as published. “GC% of *L. wadei* JMUB3933, JMUB3934, JCM16777, *Leptotrichia* sp.-1 JMUB3936, *L. shahii* JCM16776, *L. hofstadii* JCM16775, *L. trevisanii* JMUB3870, JMUB4039, JMUB3935 and *L. buccalis* C-1013-b, *Leptotrchia* sp.-3 F0260, *Leptotrichia* sp. F0590, *L. goodfellowi* JCM16774 and *Leptotrichia* sp.-6 W10393, and chromosome length of *L. wadei* JCM16777” were incorrect. The corrected Table 1 appears below.

**Table 1:** Genome and CRISPR-Cas system information of genus *Leptotrichia*.

		**Genome information**				**CRISPR/Cas class**								
**Species**	**Strain**	**Genome sequencing type^**b**^**	**Chromosome length (bp)**	**GC%**	**GenBank accession no**.	**Number of plasmid**	**Number of prophage/genomic island**	**I**	**II**	**III**	**IV**	**V**	**VI**	**Number of spacer^**c**^**
*L. wadei*	KA00185	Draft genome	n/a^a^	n/a	GCA_001553045.1	n/a	n/a	–	–	–	–	–	–	5
*L. wadei*	JMUB3933	**Complete genome**	2361227	29.6%	AP019834	0	2	I-B	–	–	–	–	VI-A1, VI-A2	47
*L. wadei*	JMUB3934	**Complete genome**	2414633	29.6%	AP019835	4	2	–	–	–	–	–	VI-A	1
*L. wadei*	F0279	Draft genome	n/a	n/a	GCA_000469405.1	n/a	n/a	–	–	–	–	–	VI-A	7
*L. wadei*	JCM16777	**Complete genome**	2305216	29.5%	AP019829	1	0	–	–	–	–	–	–	3
*L. wadei*	DSM 19758	Draft genome	n/a	n/a	GCA_000373345.1	n/a	n/a	–	–	–	–	–	–	3
*Leptotrichia* sp.-1	JMUB3936	**Complete genome**	2335974	30.1%	AP019841	3	1	–	–	III-A1, III-A2	–	–	–	22
*L. shahii*	DSM 19757	Draft genome	n/a	n/a	GCA_000373045.1	n/a	n/a	I-B	–	III-A	–	–	VI-A	51
*L. shahii*	JCM16776	**Complete genome**	2142946	29.7%	AP019827	1	1	I-B	–	III-A	–	–	VI-A	63
*Leptotrichia* sp.-2	F0557	Draft genome	n/a	n/a	GCA_000469385.1	n/a	n/a	–	–	–	–	–	VI-A	6
*L. hongkongensis*	JMUB5056	**Complete genome**	2261073	29.9%	AP019846	1	1	–	–	–	–	–	–	11
*L. massiliensis*	P3007	Draft genome	n/a	n/a	GCA_900104625.1	n/a	n/a	–	–	–	–	–	VI-A1, VI-A2	21
*L. massiliensis*	F0581	Draft genome	n/a	n/a	GCA_000469525.1	n/a	n/a	–	–	–	–	–	VI-A	10
*L. hofstadii*	JCM16775	**Complete genome**	2548198	30.6%	AP019823	3	1	–	–	III-A	–	–	–	10
*L. hofstadii*	DSM 21651	Draft genome	n/a	n/a	GCA_000428965.1	n/a	n/a	–	–	III-A	–	–	–	6
*L. hofstadii*	F0254	Draft genome	n/a	n/a	GCA_000162955.1	n/a	n/a	–	–	-	–	–	–	0
*L. trevisanii*	DSM 22070	Draft genome	n/a	n/a	GCA_000482505.1	n/a	n/a	I-B	–	III-D	–	–	–	17
*L. trevisanii*	JMUB3870	**Complete genome**	2829322	30.6%	AP019831	2	1	I-B	–	III-D	–	–	–	78
*L. trevisanii*	JMUB4039	**Complete genome**	2685755	30.8%	AP019845	0	1	I-B	–	III-D	–	–	VI-A	56
*L. trevisanii*	JMUB3935	**Complete genome**	2729392	30.6%	AP019840	0	2	-	–	III-D	–	–	–	14
*L. buccalis*	C-1013-b	Complete genome	2465610	29.6%	GCA_000023905.1	0	0	I-B	–	III-D	–	–	VI-A	102
*Leptotrichia* sp.-3	F0260	Complete genome	2194935	29.8%	GCA_001553645.1	0	2	–	–	–	–	–	–	1
*Leptotrichia* sp.-4	bin_23	Draft genome	n/a	n/a	GCA_003638725.1	n/a	n/a	–	–	–	–	–	–	0
*Leptotrichia* sp.-4	F0590	Complete genome	2152181	29.6%	GCA_002240055.1	0	1	–	–	III-D	–	–	–	37
*L. goodfellowii*	F0264	Draft genome	n/a	n/a	GCA_000176335.1	n/a	n/a	–	–	–	–	–	–	11
*L. goodfellowii*	JCM16774	**Complete genome**	2290729	31.7%	AP019822	0	1	I-B	-	III-like	–	–	–	38
*L. goodfellowii*	DSM 19756	Draft genome	n/a	n/a	GCA_000516535.1	n/a	n/a	I-B	–	III-like	–	–	–	39
*Leptotrichia* sp.-5	W9775	Draft genome	n/a	n/a	GCA_000469505.1	n/a	n/a	–	II-C	–	–	–	–	19
*Leptotrichia* sp.-6	W10393	Complete genome	2444904	31.4%	GCA_001274535.1	0	0	I-B	–	III-like	–	–	–	201

a n/a indicates not applicable;

b Bold font indicates that genome sequences were determined in this study;

c *Total number of spacer carried by all types of CRISPR-Cas systems*.

In the original article, there was an error. GC% of genome-sequenced strains was incorrect.

A correction has been made to Results and Discussion, Comparative Analysis of Leptotrichia Genome, line 373-375:

As shown in Table 1, the chromosome size of the genus *Leptotrichia* varies from 2,142,946 to 2,829,322 bp with GC contents of 29.5% to 31.7%.

The authors apologize for this error and state that this does not change the scientific conclusions of the article in any way. The original article has been updated.

